# Streamlining a Patchwork - Exploring the Challenges of Digital Transformation in Pathology: Ethnographic Study

**DOI:** 10.2196/63366

**Published:** 2025-07-18

**Authors:** Birte Linny Geisler, Ourania Amperidou, Sina Patricia Pauly, Sven Mattern, Christian M Schürch, Claudia Hermann, Christiane Stoffregen, Ilona Steinleitner, Natali Paigin, Claudia Lamß, Falko Fend, Monika A Rieger, Esther Rind, Christine Preiser

**Affiliations:** 1 Institute of Occupational and Social Medicine and Health Services Research Faculty of Medicine University Hospital Tübingen Tübingen Germany; 2 Department of Pathology and Neuropathology University Hospital and Comprehensive Cancer Center Tübingen Tübingen Germany; 3 Cluster of Excellence iFIT (EXC 2180) University of Tübingen Tübingen Germany

**Keywords:** digital pathology, digital transformation, digitization, health care, occupational health, work-related stress, ethnographic study, cultural, diversity, challenges, digital workflow, German university, pathology, employees, supervisors

## Abstract

**Background:**

To transition to a fully digital workflow, a pathology department in a German university hospital reorganized its processes, upgrading and integrating new technologies such as an updated laboratory information system and high-throughput scanners. While the visions of digital pathology follow a “promissory rhetoric” of improved patient care, studies name technological and professional challenges of digital pathology. We examined the experiences of the pathology staff with the digital transformation process from an occupational health perspective, focusing on the mutual influences between digital transformation and work-related psychosocial demands and resources.

**Objective:**

The purpose of this study was to explore the interactions between occupational health and digital transformation in the workplace based on a holistic analysis of an ongoing digital transformation process.

**Methods:**

We conducted participant observation, focus groups, qualitative interviews, and document analysis using an ethnographic research design. The pathology department had approximately 100 employees. More than 30 pathology staff members and supervisors from the diagnostics, laboratory, quality management, administration, and IT areas participated. Data were collected in 3 field phases between July 2022 and December 2023, representing different stages of the digital transformation. Data were analyzed using the reflexive thematic analysis method of Braun and Clarke. In addition, 2 member-checking workshops were conducted with the entire pathology team.

**Results:**

We identified 2 key themes and 7 subthemes. The 2 key themes were (1) highly demanding work in a complex system that does not fit and (2) striving for steadiness in an open-ended process. What we found was that the pathology department under study experienced a digital transformation process with scarce human, time, and technological resources. The results showed that the process was at the expense of the people. Digital transformation remained a compromise and did not (yet) deliver on the promise of increased work efficiency and reduced workload. The demand-resource mismatch emerged as a major digital transformation stressor. However, the slower transformation was leveraged by the pathology staff to improve the organizational culture and (again) find creative workarounds. Digital transformation led to the renegotiation of work roles and identity. It also led to the creation of a new network of connections through the implementation of new technologies but also through the creation of new forms of team communication.

**Conclusions:**

The modernization of the health care system is necessary, but it risks taking place under inadequate working conditions. Increased work intensity and perceived psychosocial stress during the transformation process threaten to drive even more people out of the health care system. Therefore, protecting the occupational health of the people implementing digital transformation should be at the core of planning digital transformation projects.

## Introduction

### Digital Transformation in Pathology

Pathology departments worldwide are moving toward digital pathology due to improvements in storage and scanner technology as well as in the field of artificial intelligence (AI) [1,2] (refer to Textbox 1 [3-5] for definitions). A key analogue practice in pathology that is now being digitized via whole-slide imaging is diagnosis. This means that, instead of examining tissue samples on glass slides under a microscope, the glass slide is scanned as a high-resolution image, and the digital slide is examined on a computer screen. Digitizing this step can be seen as a paradigm shift in the digital transformation of pathology. It affects the entire pathology workflow [6]. The implementation of digital pathology is a prerequisite for the integration of AI as the second paradigm shift in which AI-based diagnosis is expected to become central [7].

**Relevant definitions.**

**Digital transformation**

Digital transformation can be understood as “a change that occurs with the implementation of technologies” but “goes beyond digitalization as it involves changing organizational processes and tasks” [3]. Digital transformation does not focus on technology alone but considers the entire change process of an organization. New technologies lead to the emergence of “new performance, new processes, and new business models” [3]. In the health care sector, “the wide and deep use of information technologies changes how health services are delivered and processed” [3].

**Digital pathology**

Digital pathology encompasses but is not analogous to digital microscopy. Digital pathology is “a blanket term that encompasses tools and systems to digitize pathology slides and associated meta-data, their storage, review, analysis, and enabling infrastructure” [4]. Unlike traditional microscope-based pathology, digital pathology is characterized by the use of whole-slide imaging technology. By digitizing the histological slides, image analysis can now be performed on the computer screen [4]. At the level of work organization, digital pathology is “the integrated use of information technology” in pathology “to assist in the creation, sharing, and exchange of information, including data and images, and to support the complex workflow, which ranges from the receipt of study material to submission of the final data. For these purposes, digital pathology requires the development of an infrastructure that enables collaboration between different pathology facilities or health systems by allowing multimodal and multi-level pathology data to be shared by all specialists involved” [5].

The visions of digital pathology are characterized by a “promissory rhetoric” [8]. Digital pathology is expected to improve patient care, for example, through better management of high-order volumes despite staff shortages, improved diagnostic accuracy and quality control, improved collaboration among health care professionals (eg, improved data sharing for research, teaching, and multidisciplinary meetings such as tumor boards), and an increased ability of physicians to work remotely [1,9-11].

However, in contrast to the promise of better work, studies identify pitfalls of digital pathology in many steps of the process, such as misprinted barcodes, scanning errors, blurred images, and storage media failure with the risk of data loss [12-17], as well as pathologists’ concerns “to relinquish their microscope” [18]. Studies have shown that the diagnostic accuracy of whole-slide imaging is similar to conventional diagnostic performance [2,19,20]. Technical questions about the reliability of digital reporting are inevitably accompanied by work-related questions such as how certain or uncertain pathologists feel when diagnosing digitally rather than conventionally. Carboni et al [21] showed in their ethnographic study of digital pathology that digital slides and conventional glass slides require 2 different types of “professional vision” [22]. Pathologists have to learn both as they do not consider digital slides to be suitable for all cases. As a result, digital and analogue diagnostics coexist rather than mutually excluding each other [21,23].

### Digital Transformation and Work-Related Health Risks

From an occupational health perspective, potential work-related health risks of digital transformation should be discussed [24]. Studies suggest that pathology staff are affected by perceived job stress and burnout, although work in pathology is also characterized by high levels of job satisfaction. Overwhelming workloads, understaffing, additional responsibilities, and the resulting individual feelings of inadequacy and unpreparedness have been identified as important sources of perceived job stress in pathology [25-27]. Initial studies suggest that digital pathology may cause additional work-related risks, such as the risk of lower back and wrist complaints or so-called computer vision syndrome [28]. Little is known about the perceived stress faced by those who have to implement digital transformation [29]. Overall, psychosocial demands and resources have not yet played a central role in studies of digital transformation in pathology.

Concrete tools are available to address this important issue from an occupational health perspective, such as the Risk Assessment of Work-Related Psychological Stress of the Joint German Occupational Safety and Health Strategy (GDA; Gemeinsame Deutsche Arbeitsschutzstrategie) [30]. Building on established models of work-related perceived stress, the GDA scheme identifies work content, work organization, working time, social relations, work equipment, and working environment as relevant dimensions of potential psychosocial demands and resources. This helps make digital transformation tangible in terms of occupational health and break it down into its individual components. Thus, digital transformation is not an abstract process; it is made, shaped, and endured by people.

### Research Questions and Objectives

The aim of this study was to understand digital transformation in the workplace through the lens of occupational health via the example of an ongoing digital transformation process in a pathology department in Germany. While research and practice oftentimes focus on the impacts of digital transformation on work and employees’ work-related demands and resources, we also considered how working conditions in a health care system of increasingly scarce resources shape digital transformation.

Our research question was as follows: how do work-related psychosocial demands and resources and “doing” digital transformation mutually affect each other?

## Methods

### Study Design

We used a qualitative approach with an ethnographic study design. Ethnographic approaches are well suited for studying organizations [31] and their complex change processes, such as the implementation of new technologies [32]. The ethnographic approach allowed the researchers to participate in the daily work of the employees in the pathology department and to observe in detail the work activities and work experiences in different workplaces and from different perspectives. In this way, the relationship between digital transformation and work-related psychosocial demands and resources could be better understood, and new insights could be generated [33].

The research was embedded in occupational health service research [34,35]. We used the GDA’s Risk Assessment of Work-Related Psychological Stress [30] as a core sensitizing concept. Perspectives from Science and Technology Studies, particularly that of Mol [36], served as a sensitizing concept to understand the network of human and nonhuman actors and grasp digital transformation as a phenomenon that fans out into multiple digital transformations depending on the specific role and work task of each employee, as well as the transformation phase and the experiences that were already had or yet to be had [36]. Even though our analysis was more theme oriented than a solely praxeological approach would have been, the praxeological perspective helped us tackle digital transformation as a process that is enacted in the daily work routines of pathology staff.

### Study Population

This study took place in the pathology department of a German university hospital, which had approximately 100 employees and consisted of several laboratories with different testing modalities such as histopathology, immunohistology, molecular pathology, and cytology. The goal of digital transformation in this pathology department was to implement a fully digital workflow. Throughout the research process, we encountered several technology layers on the path to digital pathology (Textbox 2 [12] and Textbox 3).

**Technological levels of digital transformation in the pathology department studied.**

The digital transformation was initially led by the immunohistochemistry laboratory as a pioneer for the other laboratories in the pathology department.

**Level 1**

In the past, the department had taken first steps toward digital pathology by implementing a high-throughput scanner and small single-section scanners. Microscopes had been equipped with cameras that could also be used to scan single slides. However, the digital slides were mainly used for teaching, research, and consultation purposes, not for routine diagnosis [12]. In the meantime, these technologies became outdated.

**Level 2**

At the beginning of the research process (field phase 1; Textbox 3), the department had just implemented an updated laboratory information system suitable for digital pathology and a new labeling system, which included the use of data matrix codes with barcode scanners and an increased amount of slide printers (for details on the work tasks, refer to Multimedia Appendix 1). At that time, the changes primarily affected the administrative and laboratory staff. However, scans were already being taken using test scanners, and preparations were being made for later validation. Pathologists were already practicing digital diagnosis on-screen.

**Level 3**

The delivery of the new high-throughput scanners at the start of field phase 3 marked a turning point. The plan was to begin a crossover phase in which the digital workflow of scanning glass slides for digital diagnosis would run in parallel with the conventional workflow of microscopic diagnosis. The goal was to validate the digital workflow in the immunohistochemistry laboratory during the crossover phase to be certified by the Deutsche Akkreditierungsstelle GmbH (DAkkS), the national accreditation authority for laboratories in Germany. However, the validation of the scanners took longer than planned due to technical issues, and digital diagnosis as part of the routine workflow was still not possible at the end of the research project.

**Level 4**

The digital pathology workflow would be successfully validated and approved by the DAkkS. The pathology laboratories involved in the digital transformation project would then primarily use digital diagnosis in their routine workflow. Other laboratories in the pathology department would gradually be integrated into the digital workflow.

**Study participants and researchers by field phases.**

**Field phase 1: preparing for digital transformation**

The first phase of research took place in August 2022. At that time, technology level 2 (Textbox 2) had just been completed. In total, 2 experienced researchers carried out 5 participant observations in the main histology laboratory (where the vast amount of routine staining [mostly hematoxylin and eosin] are performed) and the immunohistochemistry laboratory (where antibody-based stains are performed) and with the pathologists during their diagnostic work (duration of 5.5, 2.75, 3, 2.75, and 1.75 hours). Participants in the first field phase were laboratory technicians and quality management (QM) staff members as well as pathologists. The researchers were CP (doctorate in sociology with many years of experience in qualitative research in occupational medicine, health service research, and criminology) and ER (PhD [health sciences], head of the research unit Healthcare for People of Working Age, and experienced in organizational and occupational health service research and qualitative research methods). The questions of interest were as follows: what is the previous and current experience of working in the pathology department for different members of staff at different workstations? What are the attitudes, expectations, and experiences of different members of this pathology department regarding digital transformation? What are the fears and hopes regarding this transformation, and what is the reality? What are the “relationships” between people and technology?

**Field phase 2: waiting for digital transformation**

The second phase of research was originally scheduled for January 2023. The idea was to study the period after the delivery of the high-throughput scanners (level 3; Textbox 2). However, due to delivery problems, the scanners did not arrive in December 2022. To avoid losing valuable time, the research team changed the research focus for field phase 2. In March 2023, an experienced researcher together with a novice researcher conducted 2 focus groups (lasting 48 and 52 minutes) and 1 semistructured qualitative interview (67 minutes) with digital transformation decision makers. Participants in the second field phase were pathologists, laboratory technicians, and IT and administrative staff with leadership roles. The researchers were BLG (doctor scientiarum humanarum with many years of experience in qualitative research in health care and professional experience in the implementation of digitalization projects) and SPP (physician with vocational training in occupational medicine, doctoral candidate, and a novice in qualitative research). The questions of interest were as follows: what are the attitudes, expectations, and experiences of decision makers regarding digital transformation? What leadership and change management concepts shape the process? How are decisions made?

**Field phase 3: arrival of digital transformation**

The third phase of research was originally scheduled to be completed no later than early summer 2023. The idea was to observe routine work with digital slides (technology level 4). However, the pathology department remained at level 3 due to continued delays in the delivery of scanners. Due to limited project resources (time, personnel, and funding), the research team shifted its research focus again. In November 2023, BLG, partly together with SPP, conducted 6 participant observations in the laboratory, diagnostics, and administration areas (duration of 2, 2.5, 3.25, 2.5, 1.5, and 2.5 hours). Participants in the third field phase were pathologists; laboratory technicians; and IT, QM, and administrative staff. The aim was to collect experiences with the moment of arrival of the long-awaited scanners in everyday work. The following questions were of interest: how is work done (differently) in pathology today? What changes does the ongoing digital transformation cause? How is the “arrival” of the scanners experienced? What influence does their presence have on attitudes, expectations, and experiences with regard to digital transformation?

The study population consisted of >30 pathology employees and supervisors from diagnostics, laboratory, quality management (QM), administration, and IT as areas of the pathology department that were relevant to digital transformation. The sample included different professional backgrounds (eg, physicians and laboratory technicians) with varying levels of work experience. Members of the multidisciplinary digital transformation core team also participated in the study. The researchers had been approached by the pathology department and asked for an accompanying occupational health study. The researchers had no previous professional ties to the pathology team. Field access was facilitated by ongoing communication with a key informant in the pathology department who kept the research team informed about the digital transformation process and helped organize data collection. Given the high workload in the pathology department, communication and scheduling were sometimes challenging, and the researchers were initially concerned about being an additional burden. However, as the research process progressed, a trusting research relationship was established between the research team and the pathology department, and the research team began to contact relevant participants within the pathology department directly, facilitating communication and scheduling.

### Ethical Considerations

This study was approved by the Ethics Committee of the Medical Faculty of the Eberhard Karls University Tübingen and the University Hospital of Tübingen in July 2022 (373/2022BO2). Before the start of field phase 1, all research participants were informed about the research project, the researchers’ role, data protection measures, and the option to opt out during staff meetings. Furthermore, informed consent was obtained either in written form or verbally during the field phases. All data were pseudonymized by BLG. Research data were stored at the Institute for Occupational and Social Medicine and Health Services Research and only accessible as a whole to the senior scientists in the project. Research participants were presented with only a small section from the data during member checks. To protect research participants further, some quotes were merged to make it more difficult to identify individual team members. The participants did not receive compensation for their participation in the study. Their study participation was part of their working time.

### Data Collection

**Figure 1:**
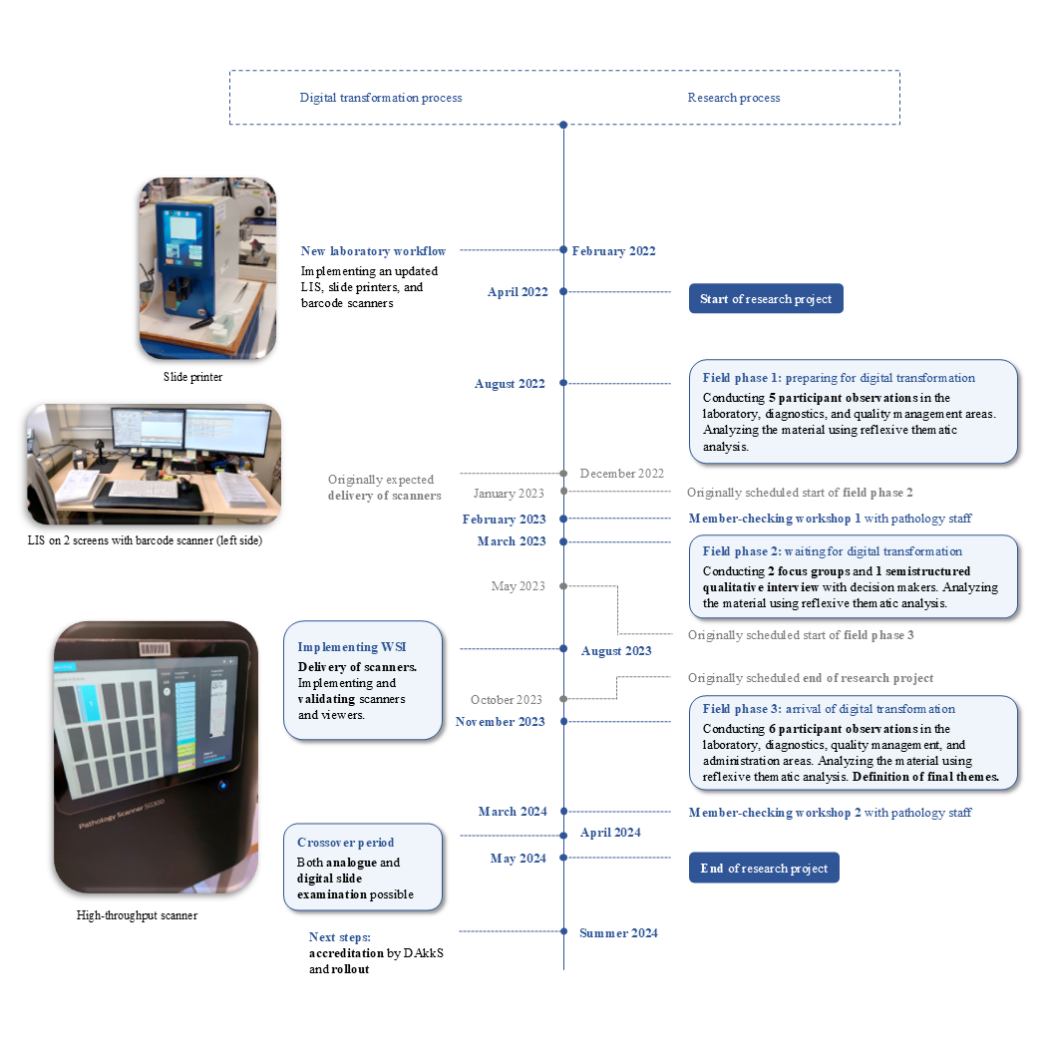
Digital transformation process and research process. DAkkS: German Accreditation Body; LIS: laboratory information system; WSI: whole-slide imaging.

As we were interested in digital transformation as a process, data collection took place in 3 field phases in August 2022, March 2023, and November 2023 (refer to the work by Bikker et al [37] for a focused ethnographic approach). Each field phase was intended to reflect relevant developmental moments in the digital transformation process. Data collection methods included participant observation using unstructured interviews; focus groups; semistructured qualitative interviews; and documents such as training documents, emails, field notes, and research diaries. Unstructured interviews during observations, the focus groups, and the semistructured interviews were audio recorded with the previous consent of the participants. Textbox 3 and Figure 1 provide details on data collection.

### Analysis

Data were transcribed by a professional office, pseudonymized by BLG, and then analyzed using the reflexive thematic analysis method by Braun and Clarke [38,39]. This method was chosen to develop core analytical themes and their relationships from the data at a level that would allow for in-depth analysis of concrete actions as well as analysis of attributed meanings and relevant contextual factors while preventing conclusions about individuals and, thus, protecting the identities of the pathology staff involved. Analysis was conducted immediately upon the completion of data collection for each field phase so that findings and open questions could be incorporated into subsequent data collection. In each phase, after familiarization with the data (eg, by reconstructing the reported “real-life” workflow; Multimedia Appendix 2), the entire dataset was coded, and initial themes were developed. Upon completion of all 3 field phases, the final themes were defined, and the findings were written up.

The analysis was a combination of individual work and group discussions. At each stage, an experienced researcher (BLG) and a student research assistant (OA; master of arts in cultural anthropology and experienced in qualitative research) analyzed the material individually. The ongoing analysis was then discussed in weekly peer debriefings by BLG, OA, and CP, always reflecting on the fact that each researcher was interpreting the data against their own professional and social background. On occasion, extended peer debriefings and journal clubs were held with the participation of SPP, ER, and MAR (medical director at the Institute for Occupational and Social Medicine and Health Services Research, occupational health physician, university professor of occupational and social medicine, and experienced in qualitative research).

In addition, 2 member-checking workshops were held, one in March 2023 (hosted by BLG, SPP, and ER) after the first phase and one after the third phase in March 2024 (hosted by BLG and ER). At the workshops, the research team presented and discussed the findings with pathology supervisors and staff. This ensured that pathology staff were able to provide their professional perspective to the analysis without access to the primary data or pseudonymized transcripts. Participants’ feedback on the findings and the research process was incorporated into the next steps of the research process [40]. Reporting of the study followed the Standards for Reporting Qualitative Research guideline [41].

## Results

### Overview

**Figure 2:**
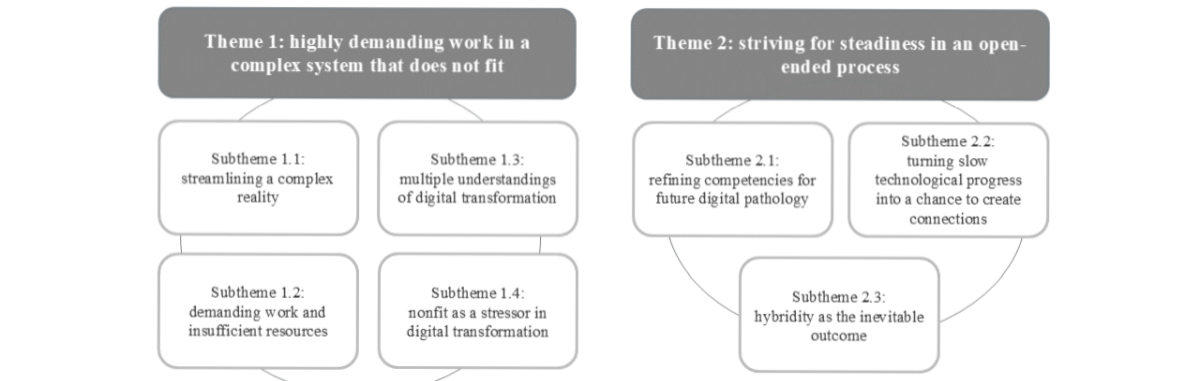
Final themes and subthemes.

We identified a distinct set of themes and subthemes in each of the 3 field phases. Figure 2 provides a visual representation of the themes that will be outlined in this section. This paper focuses on the final themes that depict the essence of the digital transformation process. Pathology is an interprofessional work setting. We differentiate between professional groups where necessary; otherwise, we speak more broadly about pathology staff.

### Theme 1: Highly Demanding Work in a Complex System That Does Not Fit

#### Overview

The work in the pathology department was characterized by complexity and high job demands in terms of speed and quality and insufficient resources in terms of time, personnel, and work equipment. It was in this overburdened context that the digital transformation of the central workflow took place. In a context already characterized by mismatch or “nonfit,” digital transformation, also characterized by nonfit, represents an additional stressor. In the following sections, we will outline the key factors that characterize the perceived digital transformation stress in pathology.

#### Subtheme 1.1: Streamlining a Complex Reality

In general, highly demanding work is a central feature of all pathology workstations (Multimedia Appendix 1) and requires smooth collaborations between humans and technologies. Laboratory technicians perform sensitive, concentrated work with their hands in interaction with microtomes, barcode scanners, slide printers, water baths, and laboratory information systems via computer screens. Pathologists perform precise diagnostic work through highly detailed viewing, searching, and describing of tissue sections against a background of broad and specific contextual knowledge and in close interaction with technology such as microscopes, computer screens, and laboratory information system. In addition, the pathology staff has to constantly check work steps and maintain an overview at each workstation to avoid errors.

In the pathology department, the work tasks in the core workflow were intertwined. This made work very complex. Although the tasks per case were linked in a linear sequence (Multimedia Appendix 1), a large number of cases had to be worked on simultaneously. This meant that the same workflow was performed in parallel for a large number of different cases. In addition, the work tasks were performed by different actors—a demanding coordination task that had to be sensitive to interfaces as mix-ups have serious implications for diagnosis and patient health. In addition, loops were often necessary, such as when pathologists requested additional staining. The case would then be returned to the laboratory, where sections would be prepared and stained again, and then returned to the diagnostic department. The fact that the pathology department was located in several buildings spread across the city added to complexities, for example, when sample tissues had to be transported back and forth and staff had to switch locations on short notice.

Apart from digital transformation, the increasing number of orders for diagnostics underlined the necessity to modernize and streamline outdated workflows. To control and manage this, comprehensive knowledge and data management were critical. This is where, for example, the laboratory information system came into play, in which each case was created and then developed as a dataset by each actor involved in the pathology process, resulting in a constant flow between data generation and data documentation. For example, the pathologists created knowledge by dictating the findings into the laboratory information system. From there, it was accessed by the administrative team, which transcribed the new audio recording if necessary and fed the information back into the laboratory information system and, thus, into the diagnostic process.

However, there was a nonfit between the complex and case-specific nature of pathology work and the standardization requirements of digital technology. Streamlining workflows impedes case-specific requirements as well as employees’ individual ways of performing work tasks, thus challenging employees’ scope for action. For example, during the second fieldwork phase, the pathologists told us about their work task of predefining fixed data fields for the structured reporting software that would be used in the future. This meant that the future writing of reports containing pathologists’ findings would be highly standardized. To define these data fields, they had to anticipate what information would be relevant and useful to their colleagues in the future based on the knowledge of the present and controlled by the options provided by the software:

Then we are still in the process of standardizing the reporting structure, which is also part of the digital transformation, into a systematic, unified reporting format with data fields. It is a lot of work to implement that and to do it in such a sustainable way that all the current regulations are considered, the current guidelines and the current knowledge. It forces us to update our own knowledge and do a lot of research. And that has nothing to do with digital transformation, it has to do with the content that needs to be stapled down. I have a responsibility there. What do I put in there? It is like a written document. I cannot change it every 2 weeks. This is an upfront effort that has to be made. I just want to get that behind me. Even though it is very exciting.

Pathologist and decision maker perspective; field phase 2; aggregated quotation

#### Subtheme 1.2: Demanding Work and Insufficient Resources

However, the conditions under which the demanding work and digital transformation took place in the pathology department were characterized by insufficient resources. We will illustrate this through the example of staff shortages and inadequate work equipment.

The increased workload, combined with a lack of sufficient staff, was compensated by the remaining staff through high work intensity; extended working time; and, during the digital transformation, unplanned extra shifts. Laboratory technicians, for example, told us that they worked longer hours so as not to overload the early shift of the following morning. Administrative staff worked longer hours so that their laboratory and diagnostic colleagues could get right back to work on cases the following day. This led to a constant feeling of work-related negative stress among pathology staff and supervisors for several years. The need for more staff in the department was so great that, in the early stages of fieldwork, the researchers were sometimes hoped to be new colleagues.

In this system already running beyond full capacity, digital transformation was perceived as an additional burden:

Going digital is a good thing. But what bothers me is that we are already so understaffed. There are too few people and too much work at the same time...and then there’s digitalization on top of it.

Laboratory staff perspective; field phase 1; aggregated quotation

In addition, the lack of adequate work equipment was an impediment to digital transformation. For example, scanners were delivered three-quarters of a year late and did not meet the technological requirements of the pathology department. The latter was in part attributed to the bidding process several years earlier, which had not considered the pathology department’s specific requirements. Another obstacle was the nonfit between the new and older work equipment, such as staining machines and glass coverslippers, which were supposed to work well with the new generation of technology but did not. The interoperability between old and new machines was characterized by disruption. For example, the glass coverslippers were so inaccurate that they caused the scanner to fail:

The scanner arm has two grippers. If we have an angled label on the glass slide and the grippers grab it, what happens? The adhesive from the slide stains the grippers. As a result, the grippers eventually become so sticky that they can no longer grip. The scanner then experiences a system failure. This has a variety of effects, including a complete system crash. We have to open the machine. Of course, this means that we have to increase the burden on the people.

Decision maker perspective; field phase 3; aggregated quotation

To compensate for the shortcomings of the technology, additional time resources had to be invested in monitoring it. With no staff to do this, even the IT project manager sometimes took on this task. Ordering new equipment to replace outdated technology proved cumbersome due to limited financial resources:

We are still using the automated glass coverslipper, which is still causing us a lot of problems today. An automated film coverslipper would be better. But then there’s the problem of funding. We need a certain amount of basic work equipment for the digital transformation. But people complain that we need it because it costs so much. So, what should we do? How can we work and change things if we don’t get the basic work equipment? It’s really depressing to be held back like that.

Laboratory staff perspective; field phase 3; aggregated quotation

What we saw here was that a lack of staff and adequate work equipment led to a mismatch between roles and work tasks and role extensions as an emergency strategy in digital transformation. The expectation that 2 generations of technology could be seamlessly combined and that the human-machine interactions could be easily replicated in the new digital process was not realistic. Instead, the pathology staff had to build the digital transformation “around” the outdated machines. This resulted in various nonfits and highlights an important discrepancy as digital transformation was supposed to increase efficiency by creating fit but did not (yet) do so:

The investment in technology does not match the ambition we have here. It’s a complete discrepancy. We’re doing Formula One here and what we have is a VW Beetle with a Ferrari engine. It just doesn’t fit together.

Decision maker perspective; field phase 3; aggregated quotation

#### Subtheme 1.3: Multiple Understandings of Digital Transformation

The perception, definition, and attribution of what digital transformation meant multiplied over time among pathology staff. It became clear that digital transformation meant different things depending on one’s responsibilities and role, the daily work content and tasks, the experience with adequate or inadequate work equipment, the level of preexisting perceived work-related stress, and how much one had already experienced working within the new workflow. For example, in the third field phase, some laboratory, QM, and administrative staff members who had already experienced many difficulties developed a negative perception of digital transformation, whereas many pathologists seemed to be more neutral. They still had little practical experience in daily work with the new digital pathology workflow because the scanners and viewers had not yet been implemented.

Digital transformation as a promise of better working conditions was mentioned less frequently over time. In the first field phase, while waiting for the next steps of digital transformation, the hopeful vision that the new technology would at least partially solve the problems of staff shortages and high workload was repeatedly expressed. Most pathology staff members viewed traditional pathology as a cumbersome way of working and looked forward to improvements through digital transformation. The scanner was latently framed as a new nonhuman colleague that could help reduce high work intensity. This was supported by the experience with early advances. For example, the implementation of barcodes and the laboratory information system led to fewer mix-ups of tissue samples. Later, laboratory technicians became increasingly fatigued by problems with the new, nonhuman “colleagues,” who did not prove to be as reliable as expected. Instead, the scanners created additional work tasks due to the extensive testing required to integrate them. Laboratory supervisors reported that they had to stay one step ahead of the technology, constantly checking to identify and correct errors and system failures. Another example is transcription. Although speech recognition of pathologists’ dictations was supposed to be a relief for the administrative staff, it actually created additional work. The transcriptionist had to constantly check and correct what the speech recognition had automatically transcribed into the system as it often made mistakes. In addition, new work tasks such as loading and monitoring the scanners and the surrounding technology proved to be monotonous and added to an already high workload. On the basis of these experiences, digital transformation was more of a burden than a solution.

In addition, in the third field phase, some staff members expressed concern about the possible loss of tasks that they liked, especially those that had to be done by hand. Here, digital transformation was viewed with sadness because it could change their job identity:

I think it’s great that I can still do things by hand. That’s why I was a bit afraid of the digital transformation. Scanning slides is fine because I still have the sectioning. That’s something you can only do by hand. And also labelling the slides...I chose this profession so that I could do something with my hands. And not so that I could just push a button. That was my interest. And that’s why I was attracted to histology, because I still have that manual work here.

Laboratory staff perspective; field phase 2; aggregated quotation

Pathologists also showed love for their manual work, such as sectioning tissue in macroscopy, and for traditional pathology instruments such as the microscope even though microscoping may cause headache or back pain sometimes:

I really like my microscope. It is a good microscope and I am very grateful for it.

Pathologist perspective; field phase 3; aggregated quotation

Before the arrival of the scanners, laboratory technicians literally ran (to) the machines. The work intensity for employees increased even more after the arrival of the scanners. This is in stark contrast to the idea that the machines should rather enable employees to perform meaningful tasks that only humans can do, such as making fine cuts on a microtome:

People run here between 6 a.m. and 8 a.m. Some people run 4 kilometers here in the morning. When you need to titrate, you hear a beep. Then you run over, titrate by hand, close the lid [of the staining machine] again, and run away. But ideally, modern technology should be sufficiently integrated into the process and developed to take that burden off these people. To give them the opportunity to realize their true destiny: To turn to their excellence.

Decision maker perspective; field phase 3; aggregated quotation

The vision of digital transformation here is to enable employees to do meaningful work based on a human-technology work relationship where technology, as a nonhuman actor, acts as an assistant to human actors to relieve them of their workload. In contrast to this vision, the reality was rather messy. Unlike the human actors, the nonhuman actors in this pathology department were unable to adapt to semioptimal conditions. They created new errors and relied on human problem-solving skills to continue working for the human actors, who in turn relied heavily on technology. The vision of a smooth work relationship between human and nonhuman actors became a rocky reality in which technology caused unpredictable disruptions to the workflow and reduced employee job control.

As a result, in the third phase of this study, digital transformation was pragmatically redefined by some pathology staff members:

The only thing we are waiting for is what will really help us. The coverslipper is giving us problems. So, we’re looking forward to a new one. The term, whether it’s digitalization, doesn’t matter. Digital transformation is just a different way of doing things. It will not make work disappear. This is also due to QM, that in a certain way it will still be the case that we have to look through everything and always keep an overview. The only difference is that now you do it on a PC and not with a glass slide in your hand. At the moment, at least in the lab, we still have the same work steps.

Laboratory staff perspective; field phase 3; aggregated quotation

Here, digital transformation was no longer seen as a source of relief but simply as a different way of performing work tasks. However, the different understandings of digital transformation did not replace each other in a linear fashion but existed in parallel. Throughout the entire process, the benefits of digital transformation were repeatedly emphasized, especially by the decision makers but also by employees. Despite the many problems in the implementation process, the vision of future pathology as a better place to work still served as a motivator:

I can imagine that we’re paying a very high price for this right now, but in the end, it will be worth it. Simply because we will be relieved in our daily lives if everything works as planned—with the AI, with the digital findings, with the synoptic reports that will come in the future and that will be fed with information from the viewer...I can imagine that the streamlining that we are doing all the time now will create a benefit in everyday life.

Pathologist and decision maker perspective; field phase 2; aggregated quotation

#### Subtheme 1.4: Nonfit as a Stressor in Digital Transformation

In our view, the discrepancies that characterized this working environment and the digital transformation process described in subthemes 1.1 to 1.3 were a central cause of the pronounced perceived work-related stress in this pathology department. The work-related stress already existed in the department independently of the digital transformation project but was significantly exacerbated as part of the digital transformation:

We’ve had this staff shortage for at least 3 or 4 years now, and of course we’ve had this stress for years...So here at work I’m so terribly tense all the time that...I’m always under electricity and I don’t feel well. And then I come home and then the burden really falls off.

Laboratory staff perspective; field phase 3; aggregated quotation

Many employees perceived psychological stress as a normal condition:

We are constantly unconsciously compensating and coping. To achieve the workload that you have to achieve, what is expected of you. And that is actually the normal situation for us. People sometimes have [physical] breakdowns.

Laboratory staff perspective; field phase 3; aggregated quotation

What seemed to be particularly stressful was that, on the one hand, the following motto—“We work for the patients”—served as a motivating guideline but, on the other hand, there was a lack of resources to meet this requirement in a healthy way. Perceiving themselves as the “guides of therapy” (pathologist perspective; field phase 3) with enormous responsibility for the life and survival of patients, pathology staff repeatedly went beyond their own limits for the sake of the patients’ health, thereby putting their own health at risk.

Coping strategies seemed limited. Pathology staff seemed to have no choice but to endure the discrepancy between the demand for optimal patient care and their actual performance. One laboratory staff member said the following:

You have to accept that not everything can be done.

There was repeated talk of perseverance as a strategy, of “just getting through it,” of “fighting through it bit by bit,” or of “keeping one’s head above water” (various voices from the laboratory; field phase 3). Some employees did not even see an increase in staff as a relief:

It’s too much work.

Pathologist perspective; field phase 3

Work-related stress, which was a normal condition in this pathology department, strongly influenced the experience of digital transformation. The feeling of being overburdened was repeatedly expressed, for example, being “overwhelmed” by digital transformation due to the lack of time resources (laboratory staff perspective; field phase 3). The mismatch created new problems that influenced the perception of what digital transformation “is”—a project fraught with problems:

What we hear is: This isn’t working yet, there are problems, this is broken, this needs to be replaced.

Laboratory staff perspective; field phase 3

The clear additional burden of digital transformation due to insufficient resources stood in stark contrast to the promise of relief through digital transformation. Decision makers seemed to be aware of this discrepancy but were unable to change it. The only thing that seemed to be under their control was the communication about it and how the “promise” was handled:

As a supervisor, you have to endure the impertinence of going through a transformation process with limited human, financial, and spatial resources, which initially means more work and more effort. Perhaps with the perspective that things will be better afterwards. You can’t promise: It’ll be done in 4 weeks and it won’t be a problem. But it would also be the wrong message to say: “It is all so difficult and I don’t know if we can do it.”

Pathologist and decision maker perspective; field phase 2; aggregated quotation

### Theme 2: Striving for Steadiness in an Open-Ended Process

#### Overview

Despite the previously mentioned lack of fit, the pathology department effectively moved forward with its digital transformation. This achievement was possible by “making it fit” and by accepting permanent nonfits. In the following sections, we will outline 3 key factors that created this compromise: refined competencies in the area of work content and work tasks (qualification) in subtheme 2.1, connectedness in the area of social relations and work organization in subtheme 2.2, and hybridity in the area of work organization in subtheme 2.3.

#### Subtheme 2.1: Refining Competencies for Future Digital Pathology

In the first phase of our study, we observed how digital transformation led to the renegotiation of work roles and identity. As we returned for the second phase of fieldwork, we identified not only changes in work practices due to the adoption of new technologies but also notable shifts in organizational culture. Organizational culture can be defined as “the shared basic assumptions, values, and beliefs” [42] that guide the way in which people act and interact, create rules, and make decisions in an organization. It includes not only principles and norms but also implicit rules and shared understandings [43]. With regard to the pathology department, we found that a specific set of competencies was increasingly emerging as a “must-have” for employees working in future digital pathology (Textbox 4).

**Competencies required of future digital pathology staff.**

- Motivation for lifelong learning and perseverance to be able to follow further processes of change and create long-term job security
- The ability as an employee to draw and defend one’s own boundaries in daily work (eg, refusing additional tasks) to remain able to work despite high and sometimes contradictory demands
- The ability to actively influence what can be influenced and accept what cannot be influenced to feel powerful despite the inevitability of certain changes
- The urge to independently develop new solutions and explore alternatives and optimizations to expand one’s sphere of influence
- Computer skills—without them, employees will not be able to work in the digital pathology of the future

The competencies mentioned by several study participants were not just skills that could be acquired but fundamental attitudes toward work and performance. These included the willingness and capacity to always do what is best for the patient, step out of one’s comfort zone, and always be open to new developments. That is, in the area of work content and work task, anyone who internalized these skills and attitudes would be suited to the digital future and would fit in digital pathology:

You have to love the job and get behind it. You have to be the “type.” I always wanted to know everything, so I kept going. I figured it all out on my own, and then things came along. Because it was never my thing to just do one thing. And I wanted to know more.

Administration staff perspective; field phase 3; aggregated quotation

It is noteworthy that the competencies needed in the future were based on competencies that already existed among the pathology staff. Therefore, this may not be so much a redefinition of competencies as a specification.

#### Subtheme 2.2: Turning Slower Technological Progress Into a Chance to Create Connections

The pathology department created a new network of connections through the implementation of new technology but also through the creation of new forms of team communication. This was not initially planned. It was rather the result of the slow pace of technological change. Contrary to expectations of a rapid digital transformation process, the transformation occurred gradually as part of a continuous exploration and adaption. While technological questions had been the initial focus of digital transformation, the focus was now shifted toward the staff. The digital transformation core team used the time “gained” to add a bottom-up decision-making culture to the existing hierarchical culture that is typical of health care. The new workflow was developed in part with all the teams involved. Employee feedback was incorporated into the technical implementation. Because of the QM requirements and the goal to be accredited as digital pathology, the digital transformation core team took the time to thoroughly test the workflow elements and optimize the human-machine interactions where possible:

For the practical implementation, it is necessary to really go into the individual teams. What are the implications? What are the details? Immunohistochemistry, for example, is very important because it is a pioneer in driving digital transformation in our department. We don’t say: Here’s what we’re going to do. Instead, we ask: Okay, what are the specific problems? And that is important. In other words, one thing is the initial decision: We are going to go this way. But the other thing is: How do we implement it? And then you really have to involve every team and listen to people, because otherwise it won’t work.

Pathologist and decision maker perspective; field phase 2; aggregated quotation

In particular, the human-to-human interactions benefited from the slower pace of technological progress. For example, the multidisciplinary digital transformation core team acted not only as a driver of change but also as a bridge to pathology staff. The core team tried to consider the needs of employees affected by digital transformation, conducted staff training, and was always available to help with technical issues. Supervisors also had time to refine their leadership role in digital transformation, such as helping their teams adapt to the new process.

Especially the teams that are understaffed are very concerned that people will have to do extra work as part of digital transformation. That’s why we in the digital transformation core team try to communicate as much as possible and try to buffer as much as possible in advance. And if there are problems, it is clear that people can always come to us. Our immunohistochemistry team in particular is very much involved in the technical side. They are all very involved. We are in direct contact with the people who will be using the new technology. So, I think it’s easy to exchange ideas with each other and the core team at virtually any time.

Pathologist and decision maker perspective; field phase 2; aggregated quote

As a result, the social relationships within the department were strengthened, which often acted as a “glue” in digital transformation. Employees told us that they held together as a team despite high levels of stress. Social relationships that had already been an important psychosocial resource in this pathology department were now expanded. However, the strong social relationships here were not characterized by constant harmony. There were also many conflicts and tensions in general and with regard to digital transformation in particular. For example, some older employees who were close to retirement decided not to participate in the change process.

Patient cases, glass slides and digital slides, diagnoses, and work tasks were increasingly connected. This is where technology began to play an important role, integrating and systematizing relevant data, work tasks, and actors in a central location. Similar to a central nervous system [44], the laboratory information system brought all the data together in one place, creating access, transparency, and oversight for each actor involved in the pathology department. For example, each glass slide could now be tracked because it now had a label with a barcode. Scanning the barcode led directly to the case in the laboratory information system. In this way, the new digital system acted as an important human-technology-human link, refining the various interactions that took place during the pathology workflow.

An administrative staff member described how the various workstations and actors involved in processing a patient’s tissue samples were mapped and linked in the laboratory information system:

Employee: So now you can see, here, the biopsy gets a number. Then it’s in this number range. [typing] Then we have it in our hands for the first time, because we have to record it. Then we get it in our hands again, because then we can write the macroscopy in here. And then it comes back to us for the microscopy.

Interviewer: So, it’s about complementing every step of the workflow that goes on in the lab.

Employee: From the lab to us, from us to the lab, from the lab to the pathologist. Wait, no, there is also the pathologist in between, right, for the macroscopy. And the zipper always has to fit.

Administration staff perspective; field phase 3; aggregated quotation

However, despite all the communication efforts, some employees still felt that they were not sufficiently informed. For example, there were repeated instances of new equipment being set up unannounced during routine work, which laboratory technicians found very stressful. Some employees did not know exactly what the current status of digital transformation was. In addition, although the digital transformation core team did everything it could to achieve the best possible compromise, digital transformation was hindered by external influences.

#### Subtheme 2.3: Hybridity as the Inevitable Outcome

The pathology staff was forced to come to terms with hybridity. It had to accept that a fully digitalized pathology workflow could not yet be realized as planned.

In the course of our observations, we noticed that a functioning system of multiple hybridities had been established in this pathology department. One example of the hybridity of analogue and digital or technology-based work was that paper remained part of the workflow. Incoming order forms were still paper based. In addition, in times of high workload and time pressure, complex documents such as QM documents and to-do lists were still printed and processed on paper. Certain QM documents still required handwritten signatures. In some cases, the need for a hybrid analogue-digital process created additional work, such as scanning the order form and transferring it to the laboratory information system. However, in some cases, there were advantages, such as the fact that paper allowed for better concentration.

**Figure 3:**
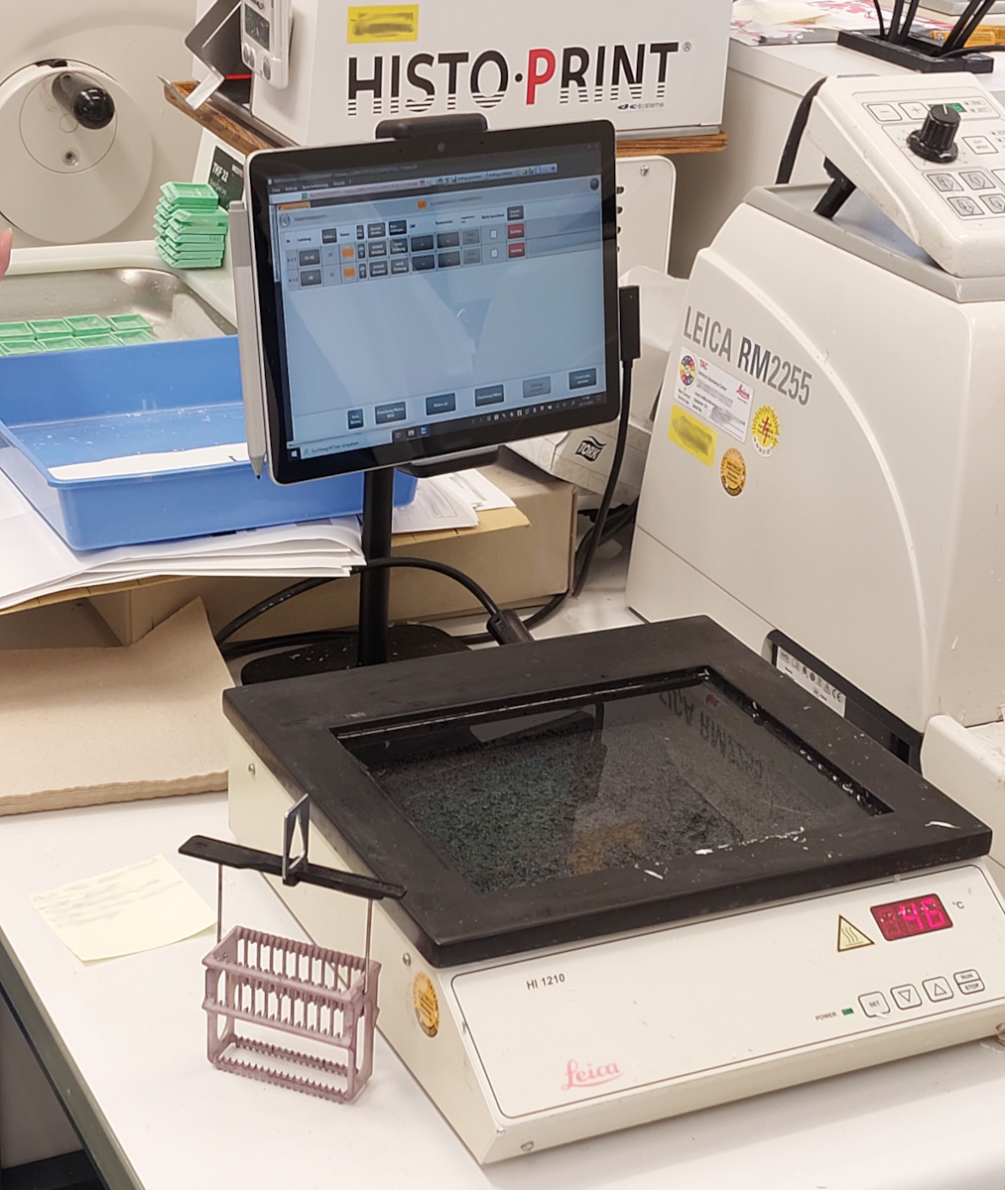
Water bath with display for case information on the left side of the microtome.

**Figure 4:**
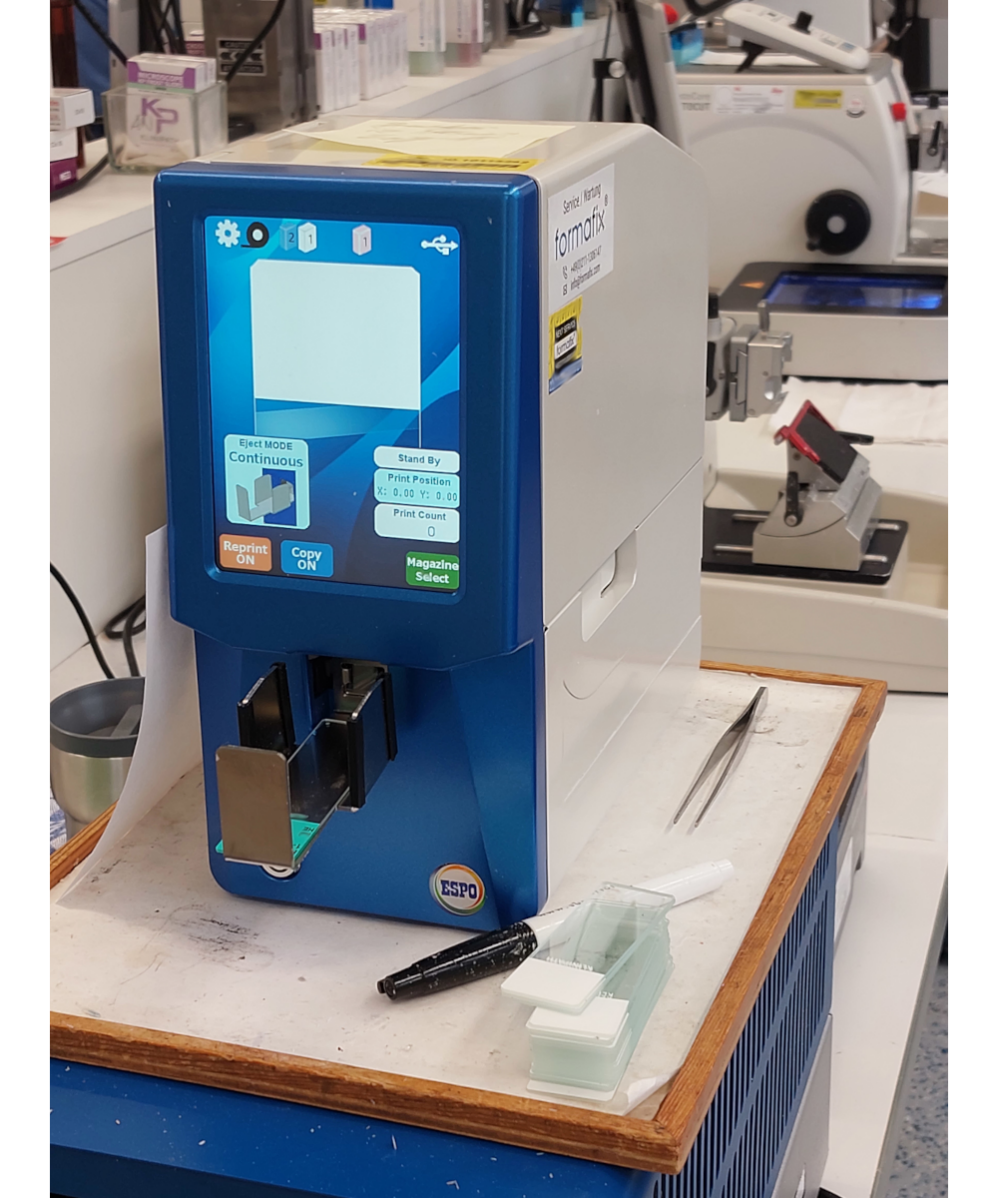
Slide printer on the right side of the microtome.

**Figure 5:**
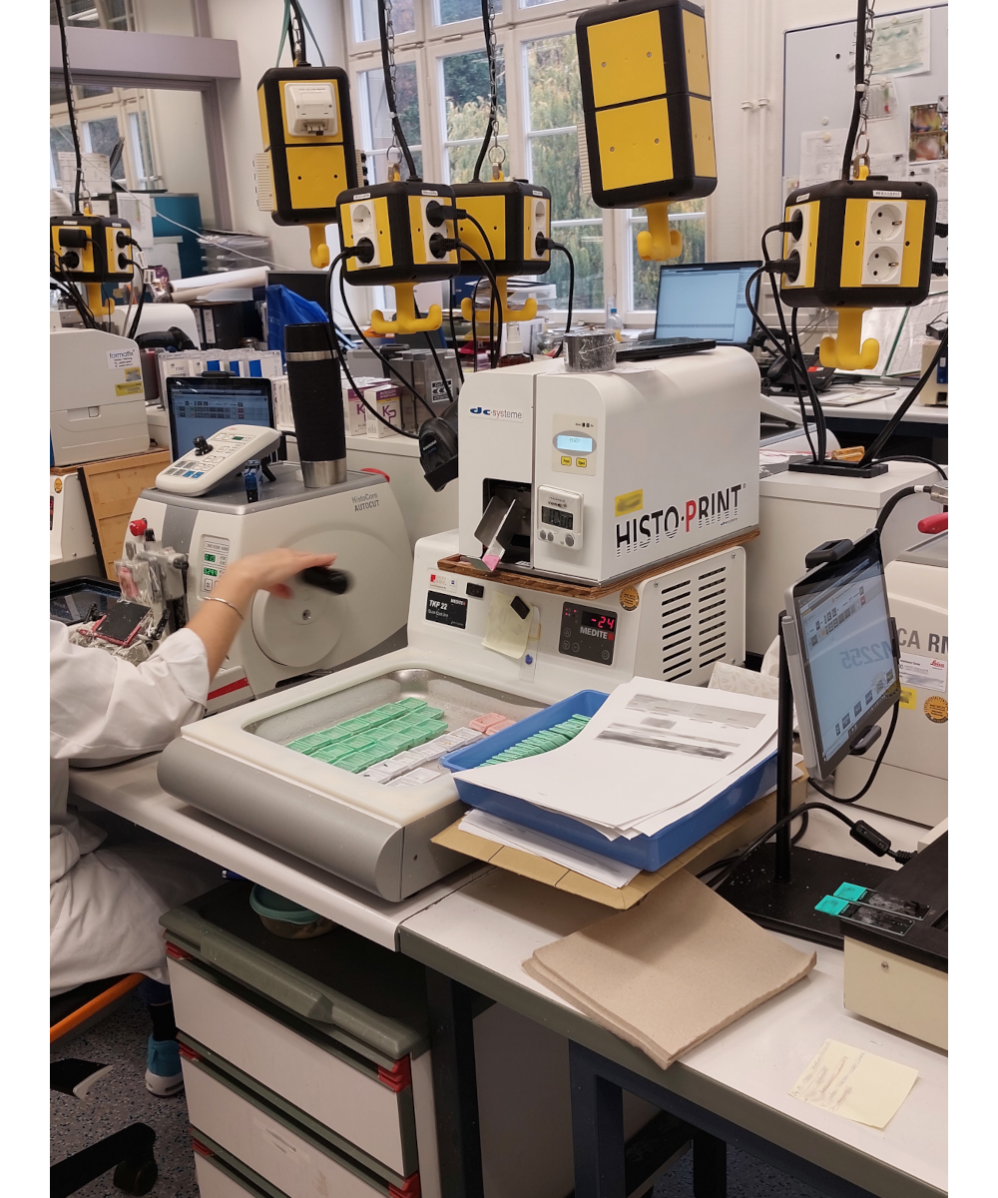
Workstation for manual sectioning of paraffin blocks with microtome.

Hybridity also increased at the human-machine interface. Analogue sectioning, for example, was performed by human pathology staff at the microtome. Now, this task was combined with digital work via computer screens mounted next to the microtome or on the slide printer (Figures 3-5). The computer screen acted as an interface between the human operator and the machine or digital system.

An example of the hybridity of individualized and standardized work steps is the digital structured reporting system. Allowing for free-text fields in addition to standardized fixed data fields served as a change strategy to “take team members along” in the digital transformation as it allowed employees to take individual paths in addition to the standard process:

It’s important to give people workarounds. There have to be “underwater pipelines” so that things that aren’t currently mapped into the system and don’t fit can still be done and don’t bring the whole process to a halt. If I have a digital mask form where data is entered, I always have to have enough free text fields where I can still be flexible about things that don’t fit into the input mask, but that need to be documented so that that information doesn’t get lost. It always has to be a hybrid of the two. So, when you set up new processes as a supervisor, you should allow people to have some individuality, which in turn makes them more willing to cooperate and accept the pre-defined things. They need to feel that their individuality still has a place here.

Pathologist and decision maker perspective; field phase 2; aggregated quotation

Although the goal was to achieve a fully digital pathology workflow, the reality was that digital transformation in pathology still could not be applied to every work task. Generally, certain work tasks in pathology will always remain analogue, such as tissue sample processing. Therefore, the pathology workflow (Multimedia Appendix 1) remains a hybrid even after digital transformation. However, it also became clear that a radical change to a digital workflow in a system where complex work processes already existed, where routine work had to continue under difficult conditions, was a fantasy. Even though the digital transformation project had not yet reached the point where digital slides were part of routine work by the third field phase of our research project, it became clear that digital transformation would never become the perfect work environment that it was supposed to be. Compromises had to be made to get people on board, such as the free-text fields. In the end, people had to fill in the gaps between the different systems, and they had to step in when the scanner was not working.

### Results From the Member-Checking Workshops

Overall, the feedback from the pathology staff at the member-checking workshops resonated with the research team’s findings. In the final workshop, several employees and supervisors even reported that they found the situation much more stressful than how the research team had portrayed it in their themes. The concept of “fit” or “nonfit” developed by the research team was particularly well received. The feedback was that, if it fit, it was because of the people in that department. A member of the digital transformation core team said the following:

The system is the reason for the mismatch, not the people. The people in pathology have to make the technology fit, because the people out there [the developers of the technology] don’t.

However, he concluded, this also means that, right now, humans are helping the machines, not the other way around.

One laboratory supervisor said that the staffing situation was currently perceived as a much greater factor of work-related stress because, in addition to understaffing, a generational change was taking place. Although it was perceived that it was much easier to train new staff members in the new process, this also meant that new staff members were unfamiliar with the “old” analogue pathology workflow and lacked important background knowledge. In the new workflow, for example, it was only necessary to “push a button” to initiate the staining process for a case. However, in situations in which the technology would not work (eg, if the equipment failed after an emergency power test), only the experienced laboratory staff members on the team would still know the analogue processes. One digital transformation core team member pointed out the following:

We still need people who know what they’re doing.

One laboratory technician worried that the background knowledge “behind the button” would be lost. Another laboratory supervisor confirmed the following:

It’s a challenge. How do we keep old knowledge up to date that is still needed? If I only have the old knowledge in theory, I may not be able to put it into practice when I need it.

As a result, the skills of pathology staff will actually need to expand because of digital transformation—in the future, it will be necessary to master both the “new” and the “old.”

What continued to motivate the pathology staff was the belief that digital pathology would eventually save time and personnel resources. One physician said the following:

But this will affect the physicians more than the laboratory technicians.

A laboratory technician remarked the following:

Technologizing is nothing new to us. We would be wrong in our profession if we had a problem with constant technological innovation.

A member of the digital transformation core team pointed out the following:

We promised: Everything will be fine. What motivates us now is the ambition to keep that promise and to correct the human-machine relationship: The human does not run for the machine, but the human has power over the machine.

## Discussion

### The Patchwork of Digital Transformations

In the ethnographic study presented in this paper, we followed a pathology department in Germany through parts of its digital transformation process from the perspective of occupational health research. Digital transformation remained a process in the making and did not deliver on the promise of increased work efficiency and reduced workload. Overall, the cost of implementing digital pathology was high, not only in terms of new hardware and software equipment but also in terms of time and personnel resources [1,10,13].

Carrying out and enduring digital transformation in the pathology department under study was a daily and rocky practice with the hope for better working conditions. Many essential decisions were made by other experts, such as the IT, administration, and hospital management areas, who had limited understanding of the practical implications [45]. For pathology staff, carrying out digital transformation was characterized by managing high workloads, coping with unmet needs, waiting for the technology to be delivered, and compensating for the lack of interoperability between technologies. The fact that old and new technologies did not fit together and had to be bridged by the pathology staff led to the emergence of illegitimate tasks [46]. Even with an advanced digital transformation process, challenges remained for pathology staff in daily work, such as the quality of digital slides [1].

While streamlining a workflow was at the center of the process, our results showed that the transformation process itself was fragmented. The process expanded over several years and remained a process in the making throughout our study. Breaking down a complex process into its details, such as work tasks and work environment, showed that digital transformation is not an all-encompassing transformation as hybridity of analogue and digital work and the coexistence of noninteroperable technologies is at the essence of the process, not just a transitional state. Carboni et al [21] point to the parallelism of 2 processes in the pathologists’ diagnosis as analogue diagnosis does not (completely) disappear. In our study, we could also see the necessity of accepting and integrating hybridity and parallelisms for other workstations in the pathology work process and, thus, complement the picture from the perspective of laboratory technicians and QM and administration staff, among others.

Simultaneously, while technology was the initial focus of digital transformation in the pathology department, the actual transformation ran deeper with regard to the social relationships and the organizational culture. Digital transformation multiplied in the sense that the assessment and definition of digital transformation can vary considerably depending on the job role, the level of influence, the level of work-related stress, and the point in time of the process. Therefore, while some of the pathology staff members and supervisors maintained an overall positive attitude toward the process, others developed more and more an attitude of pragmatic acceptance, which was accompanied by lowering expectations regarding the outcome of digital transformation and, thus, demystifying the big promise.

### Strengths and Limitations

This study focused on psychosocial aspects in the context of occupational health. In many other studies, the focus lies on the technical aspects of the transformation [13,15,16]. Occupational health–related aspects are rarely mentioned or are not fully captured. For example, the fact that pathology staff are overwhelmed by digital transformation [3] is addressed but not captured as an occupational health concept. As a result, there is a lack of consideration of the connection between working conditions and digital transformation.

What makes our study distinctive is that we looked at an entire pathology team undergoing digital transformation, focusing on the experiences of health care workers in different roles during and with digital transformation. This sets us apart from many other studies that, for example, have focused exclusively or primarily on pathologists [10,11,15,18,19]. In addition, we were involved in the process “live” over time, whereas other studies have examined transformation processes retrospectively or with a focus on the outcome [10,11]. In addition, we took an ethnographic approach and looked at the concrete work and, thus, the concrete “doing” of digital transformation in the daily lives of pathology staff. This approach and all these components allowed us to observe multiplicities.

On the other hand, the definition of the field phases and the strong focus on “events” such as the arrival of the scanners sometimes hindered the research process. Thus, during delays in the delivery of the core technology (arrival of the scanners), the research team became cowaiters [47]. An alternative approach would have been to follow the department continuously, for example, by visiting them once a month. However, the ability to reflect and partially adjust the research focus demonstrated the strength of the chosen method. Ethnography consistently follows the real-life situation, whatever it may be. This makes it suitable for studying unpredictable and complex change processes such as digital transformation.

### Promissory Rhetoric and the Reality of Work-Related Demands and Resources

Our findings add to those of other studies that address the expectations of digital transformation that are shaped and driven by a “promissory rhetoric” [8], in which current pressing issues (such as staff shortages) are promised to be solved once the process is complete [8,48]. Kusta et al [48] have shown that the promise of digital transformation serves political and strategic purposes, whereas the reality of those carrying out digital transformation in their daily practice does not live up to this promise. Our results emphasize that the process of digital transformation comes at the expense of people in their daily work. According to the dimensions defined for the “Consideration of psychosocial factors in risk assessment” of the GDA [30], work-related stress in this pathology department occurred mainly in the area of work organization (working time, work intensity, disruptions and interruptions, and communication and cooperation) and in the area of work content and work tasks (completeness of tasks, job control, and information). In the area of working environment, work-related stressors related to noise (eg, caused by the staining machines), and physical factors (eg, having to walk between machines and work tasks in the early shift). Workplace design, information design, and work equipment also played a role.

In the pathology department under study, digital transformation created incomplete and messy technologies that sometimes fulfilled the promise of streamlined and efficient workflows and sometimes did not. Rather, digital transformation drew additional attention to underlying issues such as preexisting staff shortages that shaped the implementation process. The already existing nonfit faced by pathology staff also characterized the digital transformation process—doing demanding work but without sufficient resources and implementing new technologies that require standardization of work processes, whereas the reality of work was influenced by constant efforts to compensate for staff shortages and technology failures. Although the idea was that digital transformation would streamline the work process and make work more efficient, the pathology staff had to constantly find creative workarounds to make digital transformation work, such as the IT project manager supporting the laboratory technicians on the first laboratory shift because the new slide scanner was not working as expected. The pathology team solved this by breaking new ground on a work culture level and simply “making the best of it.” This included accepting that digital transformation was in fact an open-ended hybrid process. From an occupational health perspective, all of this led to significant perceived psychological stress [30] among pathology staff. A “normality of work overload” became apparent in this department [49]. Our findings suggest that digital transformation may lead to an additional burden for health care workers and may intensify already existing high job demands [50,51].

According to the demand-control model, high job demands can be partially compensated by high job control, whereas high job demands with low job control can have negative effects on employees’ health [52,53]. The coexistence of digital transformation and the unpredictably long wait for the next steps led to a strain on the employees in the department under study. In addition, the dependence on external stakeholders such as the hospital’s IT department and external suppliers made digital transformation a stressful experience. This is also reported in other studies [45]. The need of people to stay in control—especially when experiencing technology to be unreliable—led to the continuous coexistence of digital and paper-based documentation because paper-based documents in some steps of the process were perceived as more reliable. This has been reported in various health care settings [45,54]. We observed that the pathology staff managed to continue the rocky digital transformation process by repeatedly regaining job control using the waiting period to actively refine their organizational culture and the competencies needed for future digital pathology [55].

Conducting digital transformation in the pathology department was characterized by a highly motivated attitude toward an exhausting process as successfully achieved intermediate steps repeatedly generated a sense of achievement and new hope—at least for some of the pathology staff members involved. From an occupational health perspective, it is interesting to note that, for some pathology staff members, the promise of technology improving working conditions seemed to have been powerful enough to make them accept additional work-related perceived stress. Of course, health care workers are already used to high workloads and work-related perceived stress, which may make them particularly resilient to even higher levels of perceived stress. Other members of the pathology department were more motivated by the idea of “getting it done.” They seemed to let go of the promise of a better workplace.

In addition, the digital transformation core team played an important role in mitigating the work-related perceived stress caused by digital transformation. This is similar to findings of other studies that identified social relationships as a core psychosocial resource for coping with work-related perceived stress among health care workers [51,56] and studies on digital transformation in pathology that emphasized the creation of a multidisciplinary project team with not only technical skills but also communication and change management skills [14,15]. The digital transformation core team identified and communicated the requirements in their teams, developed new workflows, mediated between the core digital transformation team and their own staff, and integrated new staff into the renewed processes. In this way, they were trying to keep their team fit for work—often at their own expense.

### Implication for Practice

Perceived psychosocial stress during digital transformation risks driving even more employees out of the health care system [51,57,58]. Practical implications to address these issues can be considered at the macro, meso, and micro levels.

At the *macro level (the health care system)*, the shortage of health care workers is an important problem when it comes to managing complex digital transformation processes over a long period in addition to routine work. Health care workers in Germany already experience alarming levels of work-related perceived stress [51]. Despite high medical standards, Germany has a low number of pathologists compared to other high-income countries [59]. This has implications for the practical implementation of a digital transformation project at the *meso level (the hospital)*. Digital transformation should not come at the expense of health care workers. Therefore, before embarking on a digital transformation process, working conditions should be evaluated, and personnel resources should be adapted to the needs of the transformation project—which is limited by the underlying problem at the macro level. At the *micro level (the pathology department)*, the consequences of changing job roles should be considered. For example, the job of laboratory technician may become less attractive if it involves taking over the extensive scanning tasks [60]. To help employees regain job control, supervisors should enable their employees to hone competencies [3] that prepare them for the transformation. Social relationships should be strengthened as an important work-related psychosocial resource to balance the high job demands in the health care sector in general [61] and of digital transformation in particular [51]. Nevertheless, the importance of job control and social relationships as psychosocial factors should not gloss over the fact that the most pressing sources of health care workers’ perceived work-related stress need to be addressed at the macro level.

### Conclusions

The necessary modernization of the health care system is at risk of taking place under inadequate working conditions. Protecting the occupational health of the people who make digital transformation possible should be at the heart of planning digital transformation projects. For pathology, this is especially important in view of the next stage of digital transformation, the implementation of AI [62-65].

## Appendix

Multimedia Appendix 1 Routine workflow in the pathology department under study.

Multimedia Appendix 2 Reconstruction of the “real-life” workflow in the pathology department in field phase 1.

## Abbreviations

AI: artificial intelligence
GDA: Joint German Occupational Safety and Health Strategy (Gemeinsame Deutsche Arbeitsschutzstrategie)
QM: quality management

## References

[1] Pinto DG, Bychkov A, Tsuyama N, Fukuoka J, Eloy C (2023). Real-world implementation of digital pathology: results from an intercontinental survey. Lab Invest.

[2] Rizzo PC, Caputo A, Maddalena E, Caldonazzi N, Girolami I, Dei Tos AP, Scarpa A, Sbaraglia M, Brunelli M, Gobbo S, Marletta S, Pantanowitz L, Della Mea V, Eccher A (2023). Digital pathology world tour. Digit Health.

[3] Reis J, Melão N (2023). Digital transformation: a meta-review and guidelines for future research. Heliyon.

[4] Abels E, Pantanowitz L, Aeffner F, Zarella MD, van der Laak J, Bui MM, Vemuri VN, Parwani AV, Gibbs J, Agosto-Arroyo E, Beck AH, Kozlowski C (2019). Computational pathology definitions, best practices, and recommendations for regulatory guidance: a white paper from the Digital Pathology Association. J Pathol.

[5] Pallua JD, Brunner A, Zelger B, Schirmer M, Haybaeck J (2020). The future of pathology is digital. Pathol Res Pract.

[6] Zarella MD, Bowman D, Aeffner F, Farahani N, Xthona A, Absar SF, Parwani A, Bui M, Hartman DJ (2019). A practical guide to whole slide imaging: a white paper from the digital pathology association. Arch Pathol Lab Med.

[7] Reis-Filho JS, Kather JN (2023). Overcoming the challenges to implementation of artificial intelligence in pathology. J Natl Cancer Inst.

[8] Stevens M, Wehrens R, Kostenzer J, Weggelaar-Jansen AM, de Bont A (2022). Why personal dreams matter: how professionals affectively engage with the promises surrounding data-driven healthcare in Europe. Big Data & Society.

[9] Koelzer VH, Grobholz R, Zlobec I, Janowczyk A, Swiss Digital Pathology Consortium (SDiPath) (2021). Update on the current opinion, status and future development of digital pathology in Switzerland in light of COVID-19. J Clin Pathol.

[10] Kelleher M, Colling R, Browning L, Roskell D, Roberts-Gant S, Shah KA, Hemsworth H, White K, Rees G, Dolton M, Soares MF, Verrill C (2023). Department wide validation in digital pathology-experience from an academic teaching hospital using the UK Royal College of Pathologists' guidance. Diagnostics (Basel).

[11] Patterson ES, Mansour L, Gurcan MN, Li Z, Parwani A (2019). Predicting opportunities and challenges prior to transitioning to digital pathology: an interview envisioning study with 11 pathologists. Proc Int Symp Hum Factors Ergon Health Care.

[12] Mattern S, Schürch C (2022). Einführung eines komplett digitalisierten Workflows für die Pathologie am Universitätsklinikum Tübingen. Trillium Digitale Pathologie.

[13] Dawson H (2022). Digital pathology - rising to the challenge. Front Med (Lausanne).

[14] Fraggetta F, L'Imperio V, Ameisen D, Carvalho R, Leh S, Kiehl T, Serbanescu M, Racoceanu D, Della Mea V, Polonia A, Zerbe N, Eloy C (2021). Best practice recommendations for the implementation of a digital pathology workflow in the anatomic pathology laboratory by the European Society of Digital and Integrative Pathology (ESDIP). Diagnostics (Basel).

[15] Stathonikos N, Nguyen TQ, Spoto CP, Verdaasdonk MA, van Diest PJ (2019). Being fully digital: perspective of a Dutch academic pathology laboratory. Histopathology.

[16] Cheng CL, Azhar R, Sng SH, Chua YQ, Hwang JS, Chin JP, Seah WK, Loke JC, Ang RH, Tan PH (2016). Enabling digital pathology in the diagnostic setting: navigating through the implementation journey in an academic medical centre. J Clin Pathol.

[17] Schwen LO, Kiehl TR, Carvalho R, Zerbe N, Homeyer A (2023). Digitization of pathology labs: a review of lessons learned. Lab Invest.

[18] Bruce C, Prassas I, Mokhtar M, Clarke B, Youssef E, Wang C, Yousef GM (2024). Transforming diagnostics: the implementation of digital pathology in clinical laboratories. Histopathology.

[19] Baidoshvili A, Khacheishvili M, van der Laak JA, van Diest PJ (2023). A whole-slide imaging based workflow reduces the reading time of pathologists. Pathol Int.

[20] Kusta O, Rift CV, Risør T, Santoni-Rugiu E, Brodersen JB (2022). Lost in digitization - a systematic review about the diagnostic test accuracy of digital pathology solutions. J Pathol Inform.

[21] Carboni C, Wehrens R, van der Veen R, de Bont A (2023). Eye for an AI: more-than-seeing, fauxtomation, and the enactment of uncertain data in digital pathology. Soc Stud Sci.

[22] Goodwin C (1994). Professional vision. Am Anthropol.

[23] Thorstenson S, Molin J, Lundström C (2014). Implementation of large-scale routine diagnostics using whole slide imaging in Sweden: digital pathology experiences 2006-2013. J Pathol Inform.

[24] Howard A, Antczak R, Albertsen K (2022). Third European survey of enterprises on new and emerging Risks (ESENER 2019): overview report. European Agency for Safety and Health at Work (EU-OSHA).

[25] Kelly M, Soles R, Garcia E, Kundu I (2020). Job stress, burnout, work-life balance, well-being, and job satisfaction among pathology residents and fellows. Am J Clin Pathol.

[26] Garcia E, Kundu I, Kelly M, Soles R, Mulder L, Talmon G (2020). The American Society for Clinical Pathology's job satisfaction, well-being, and burnout survey of laboratory professionals. Am J Clin Pathol.

[27] Khan S (2024). The dark side of being a pathologist: unravelling the health hazards. Indian J Pathol Microbiol.

[28] Jahn SW, Plass M, Moinfar F (2020). Digital pathology: advantages, limitations and emerging perspectives. J Clin Med.

[29] Frennert S (2023). Moral distress and ethical decision-making of eldercare professionals involved in digital service transformation. Disabil Rehabil Assist Technol.

[30] Beck D, Taskan E, Elskamp E, Gold M, Gregersen S, Klamroth H, Mields J, Sandrock S, Schuller K, Thorein A, Tiedemann MB, Willingstorfer B, Wittmann S (2022). Consideration of psychosocial factors in risk assessment: recommendations for implementation in business practice. Gemeinsame Deutsche Arbeitsschutzstrategie (GDA).

[31] Kostera M, Harding N (2021). Organizational Ethnography.

[32] Gertner AK, Franklin J, Roth I, Cruden GH, Haley AD, Finley EP, Hamilton AB, Palinkas LA, Powell BJ (2021). A scoping review of the use of ethnographic approaches in implementation research and recommendations for reporting. Implement Res Pract.

[33] Lähdesmäki T, Koskinen-Koivisto E, Čeginskas VL, Koistinen AK (2020). Challenges and Solutions in Ethnographic Research: Ethnography with a Twist.

[34] Sears JM, Wickizer TM, Franklin GM, Fulton-Kehoe D, Hannon PA, Harris JR, Graves JM, McGovern PM (2023). Development and maturation of the occupational health services research field in the United States over the past 25 years: challenges and opportunities for the future. Am J Ind Med.

[35] Ansmann L, Nöst S, Körner M, Auschra C, Bal R, Böddeker M, Bode I, Braithwaite J, Breidenbach C, Coors M, Demirer I, Exworthy M, Harst L, Heuser C, Hoffmann J, Köberlein-Neu J, Krajic K, Maniatopoulos G, Mannion R, Möhler R, Pfaff H, Rieger MA, Rind E, Helge Schnack MA, Anke Wagner MA, Weigl M, Wensing M, Wiig S, Wild E, Wilhelm H, Wirtz M, Götz K (2024). Correction: navigating the future of organisational health services research in Germany and beyond: a position paper. Gesundheitswesen.

[36] Mol A (2002). The Body Multiple: Ontology in Medical Practice.

[37] Bikker AP, Atherton H, Brant H, Porqueddu T, Campbell JL, Gibson A, McKinstry B, Salisbury C, Ziebland S (2017). Conducting a team-based multi-sited focused ethnography in primary care. BMC Med Res Methodol.

[38] Braun V, Clarke V (2022). Thematic Analysis: A Practical Guide.

[39] Braun V, Clarke V (2019). Reflecting on reflexive thematic analysis. Qual Res Sport Exerc Health.

[40] Birt L, Scott S, Cavers D, Campbell C, Walter F (2016). Member checking: a tool to enhance trustworthiness or merely a nod to validation?. Qual Health Res.

[41] O'Brien BC, Harris IB, Beckman TJ, Reed DA, Cook DA (2014). Standards for reporting qualitative research: a synthesis of recommendations. Acad Med.

[42] Schneider B, Ehrhart MG, Macey WH (2013). Organizational climate and culture. Annu Rev Psychol.

[43] Schein EH (2016). Organizational Culture and Leadership. 5th edition.

[44] Rovira-Simón J, Sales-I-Coll M, Pozo-Rosich P, Gates D, Patt C, Hennessey I, Emery L, Hueto-Madrid JA, Carbonell-Cobo M, Garcia-Cuyàs F, Moz M, Chaudry Z, Shaw G (2022). Introduction to the cognitive hospital. Future Healthc J.

[45] Choroszewicz M (2023). (In)visible everyday work of fostering a data‐driven healthcare and social service organisation. New Technol Work Employ.

[46] Semmer NK, Jacobshagen N, Meier LL, Elfering A, Kälin W, Tschan F, Bundesanstalt für Arbeitsschutz und Arbeitsmedizin, Junghanns G, Morschhäuser M (2013). Psychische Beanspruchung durch illegitime Aufgaben. Immer schneller, immer mehr: Psychische Belastung bei Wissens- und Dienstleistungsarbeit.

[47] Preiser C, Geisler BL, Amperidou O, Rind E, Rieger MA (2024). "Living Gantt-Chart" – methodical reflection of waiting and flexible adaptation to practice in an ethnographic research process. German Medical Science.

[48] Kusta O, Bearman M, Gorur R, Risør T, Brodersen JB, Hoeyer K (2024). Speed, accuracy, and efficiency: the promises and practices of digitization in pathology. Soc Sci Med.

[49] Kratzer N, Dunkel W, Bundesanstalt für Arbeitsschutz und Arbeitsmedizin, Junghanns G, Morschhäuser M (2013). Neue Steuerungsformen bei Dienstleistungsarbeit – Folgen für Arbeit und Gesundheit. Immer schneller, immer mehr: Psychische Belastung bei Wissens- und Dienstleistungsarbeit.

[50] Adam D, Berschick J, Schiele JK, Bogdanski M, Schröter M, Steinmetz M, Koch AK, Sehouli J, Reschke S, Stritter W, Kessler CS, Seifert G (2023). Interventions to reduce stress and prevent burnout in healthcare professionals supported by digital applications: a scoping review. Front Public Health.

[51] Worringer B, Genrich M, Müller A, Gündel H, Angerer P, Contributors of the Seegen Consortium (2020). Hospital medical and nursing managers' perspective on the mental stressors of employees. Int J Environ Res Public Health.

[52] Seidler A, Schubert M, Freiberg A, Drössler S, Hussenoeder FS, Conrad I, Riedel-Heller S, Starke KR (2022). Psychosocial occupational exposures and mental illness. Dtsch Arztebl Int.

[53] Karasek RA (1979). Job demands, job decision latitude, and mental strain: implications for job redesign. Adm Sci Q.

[54] Canfell OJ, Meshkat Y, Kodiyattu Z, Engstrom T, Chan W, Mifsud J, Pole JD, Byrne M, Raders EV, Sullivan C (2022). Understanding the digital disruption of health care: an ethnographic study of real-time multidisciplinary clinical behavior in a new digital hospital. Appl Clin Inform.

[55] Krzywdzinski M, Butollo F (2022). Combining experiential knowledge and artificial intelligence. The digital transformation of a traditional machine-building company. MRev.

[56] Meyer SC, Tisch A (2024). Exploring the relationship between techno-unreliability at work and burnout. J Occup Environ Med.

[57] Bäckström J, Pöder U, Karlsson AC (2024). I was merely a brick in the game: a qualitative study on registered nurses' reasons for quitting their jobs in hospitals. J Nurs Manag.

[58] Mambrey V, Dreher A, Loerbroks A (2024). Leaving the profession as a medical assistant: a qualitative study exploring the process, reasons and potential preventive measures. BMC Health Serv Res.

[59] Märkl B, Füzesi L, Huss R, Bauer S, Schaller T (2021). Number of pathologists in Germany: comparison with European countries, USA, and Canada. Virchows Arch.

[60] Jensen CL, Thomsen LK, Zeuthen M, Johnsen S, El Jashi R, Nielsen MF, Hemstra LE, Smith J (2024). Biomedical laboratory scientists and technicians in digital pathology - is there a need for professional development?. Digit Health.

[61] Schön Persson S, Nilsson Lindström P, Pettersson P, Andersson I, Blomqvist K (2018). Relationships between healthcare employees and managers as a resource for well-being at work. Society Health Vulnerability.

[62] Zarella MD, McClintock DS, Batra H, Gullapalli RR, Valante M, Tan VO, Dayal S, Oh KS, Lara H, Garcia CA, Abels E (2023). Artificial intelligence and digital pathology: clinical promise and deployment considerations. J Med Imag.

[63] Drogt J, Milota M, Vos S, Bredenoord A, Jongsma K (2022). Integrating artificial intelligence in pathology: a qualitative interview study of users' experiences and expectations. Mod Pathol.

[64] Sarwar S, Dent A, Faust K, Richer M, Djuric U, Van Ommeren R, Diamandis P (2019). Physician perspectives on integration of artificial intelligence into diagnostic pathology. NPJ Digit Med.

[65] Tizhoosh HR, Pantanowitz L (2018). Artificial intelligence and digital pathology: challenges and opportunities. J Pathol Inform.

