# Supplementary material for: Streamlining a Patchwork - Exploring the Challenges of Digital Transformation in Pathology: Ethnographic Study
Source: J Med Internet Res. 2025 Jul 18;27:e63366. doi: 10.2196/63366 (PMC12317291; doi:10.2196/63366)
Supplement: Multimedia Appendix 1 [file jmir_v27i1e63366_app1.docx]

## Multimedia Appendix 2: Routine workflow in the pathology department under study

| **Work tasks** | **Description of work task** | **Impact of digital transformation** |
| --- | --- | --- |
| **1. Tissue sample reception** | - Tissue sample arrives at the laboratory in formalin-filled containers, along with a paper-based order form from clinicians - Case registration by laboratory or administrative staff | - Creating a case in the digital laboratory information system - Scanning the order form and associating it with the case - Generating a case-specific QR code on labels and applying the labels to the order and the tissue samples   Main change: Cases are now numbered in the order they are created. In addition, the linking of capsules and slides to the case, including assignment to the respective sample container within the case, was introduced with the adaptation of the workflow. |
| **2. Macroscopy and sectioning** | - Gross processing of specimen by pathologists - Sectioning of material and selection of diagnostically relevant tissue areas using vision and touch - Placement in capsules - Documentation of observations | - Documentation via dictation using a foot pedal - Direct transfer to the laboratory information system and assignment to the case - Via screens, access to the laboratory information system and all relevant case information, as well as to the QM system, which contains the organ-specific standard process |
| **3. Tissue sample preparation** | - Dehydration and paraffin-embedding of tissue samples - Cutting of wafer-thin sections and mounting on glass slides - Application of specific stains - Performed by laboratory technicians using a variety of equipment such as staining machines and microtomes | - Use of slide printers to print the case-related data matrix code on the glass slides - Use of the laboratory information system to view special staining requirements and case-related information relevant to tissue sectioning (eg, if bone is to be sectioned, which requires sharper blades in the microtome) |
| **4. Case assignments** | - Collection of all glass slides from a case and sorting into case folders by laboratory technicians - Delivery of folders to pathologists | - Scanning of glass slides using high-throughput scanners - Using the laboratory information system to automatically assign digital slides to cases - Using the laboratory information system to send digital slides to pathologists |
| **5. Diagnosis** | - Microscopic examination of slides and documentation of findings by pathologists - If further examination is required, additional sectioning and staining may be ordered at the laboratory - In this case, repetition of steps 3 and 4 in the laboratory | - Use of the laboratory information system to access case information relevant to the diagnosis - Dictation documentation in the laboratory information system using speech recognition for the majority of reports (especially foreground speech recognition, with direct approval without the need for administrative staff intervention) - In the future: examinations only on screen using viewer software |
| **6. Writing and mailing of diagnostic report** | - Writing of final diagnostic reports based on documented case information by transcription or billing staff - After approval by an attending pathologist, delivery of reports to clinicians - Generating invoices - In case of external practices as clients, possible need to send report and invoices as paper letter | - Mailing of reports to approving pathologists and approval through the laboratory information system - Digital delivery of final reports to clinicians - Billing through the clinical information system, which is still a separate system and therefore not aligned with the laboratory information system |
| **7. Archiving** | - Archiving of paraffin-embedded tissue specimen blocks and glass slides by laboratory staff - Storing and archiving of specimens, e.g. when follow-up is required, by systematically sorting glass slides into boxes and storing them in cabinets | In the future:   - Digital archiving of digital slides - Performing the sorting process by laboratory staff without the need to collect slides from pathologists - However: Since glass slides are legally required to be stored for ten years, they will continue to be stored. |
