# Supplementary figures and images for: Streamlining a Patchwork - Exploring the Challenges of Digital Transformation in Pathology: Ethnographic Study
Source: J Med Internet Res. 2025 Jul 18;27:e63366. doi: 10.2196/63366 (PMC12317291; doi:10.2196/63366)

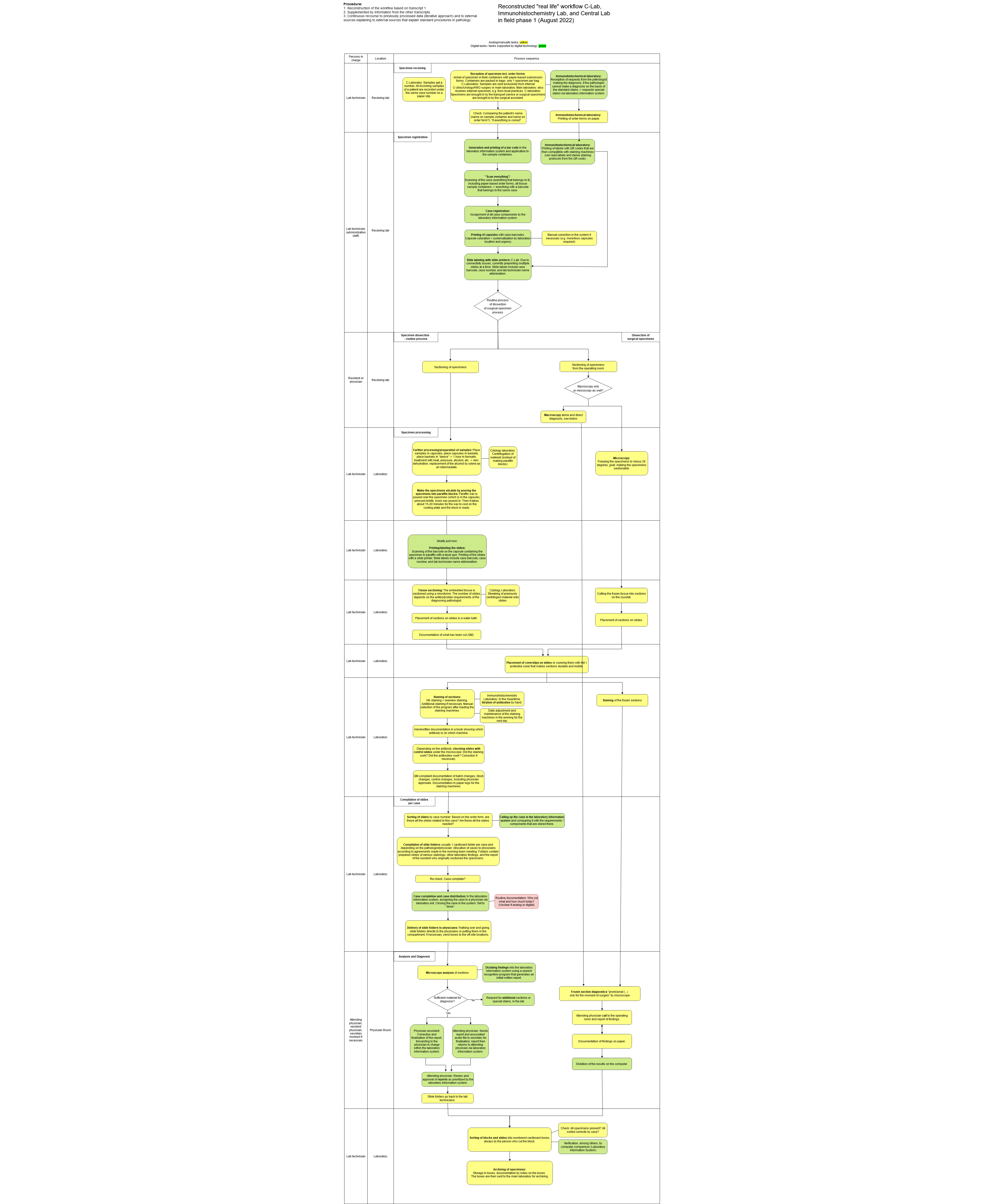

Supplement: Multimedia Appendix 2 [file jmir_v27i1e63366_app2.png]
